# Combined Optimization of Codon Usage and Glycine Supplementation Enhances the Extracellular Production of a β-Cyclodextrin Glycosyltransferase from *Bacillus* sp. NR5 UPM in *Escherichia coli*

**DOI:** 10.3390/ijms21113919

**Published:** 2020-05-30

**Authors:** Nik Ida Mardiana Nik-Pa, Mohamad Farhan Mohamad Sobri, Suraini Abd-Aziz, Mohamad Faizal Ibrahim, Ezyana Kamal Bahrin, Noorjahan Banu Mohammed Alitheen, Norhayati Ramli

**Affiliations:** 1 Department of Bioprocess Technology, Faculty of Biotechnology and Biomolecular Sciences, Universiti Putra Malaysia, UPM Serdang 43400, Selangor, Malaysia; nikmardiana@unikl.edu.my (N.I.M.N.-P.); mfarhan@unimap.edu.my (M.F.M.S.); suraini@upm.edu.my (S.A.-A.); faizal_ibrahim@upm.edu.my (M.F.I.); ezyana@upm.edu.my (E.K.B.); 2 Section of Bioengineering Technology, University Kuala Lumpur Branch Campus Malaysian Institute of Chemical & Bioengineering Technology, Taboh Naning, Alor Gajah 78000, Melaka, Malaysia; 3 School of Bioprocess Engineering, Universiti Malaysia Perlis, Kompleks Pusat Pengajian Jejawi 3, Arau 02600, Perlis, Malaysia; 4 Department of Cell and Molecular Biology, Faculty of Biotechnology and Biomolecular Sciences, Universiti Putra Malaysia, UPM Serdang 43400, Selangor, Malaysia; noorjahan@upm.edu.my

**Keywords:** cyclodextrin glycosyltransferase, codon usage, cyclodextrin, glycine, inducer, extracellular enzyme

## Abstract

Two optimization strategies, codon usage modification and glycine supplementation, were adopted to improve the extracellular production of *Bacillus* sp. NR5 UPM β-cyclodextrin glycosyltransferase (CGT-BS) in recombinant *Escherichia coli*. Several rare codons were eliminated and replaced with the ones favored by *E. coli* cells, resulting in an increased codon adaptation index (CAI) from 0.67 to 0.78. The cultivation of the codon modified recombinant *E. coli* following optimization of glycine supplementation enhanced the secretion of β-CGTase activity up to 2.2-fold at 12 h of cultivation as compared to the control. β-CGTase secreted into the culture medium by the transformant reached 65.524 U/mL at post-induction temperature of 37 °C with addition of 1.2 mM glycine and induced at 2 h of cultivation. A 20.1-fold purity of the recombinant β-CGTase was obtained when purified through a combination of diafiltration and nickel-nitrilotriacetic acid (Ni-NTA) affinity chromatography. This combined strategy doubled the extracellular β-CGTase production when compared to the single approach, hence offering the potential of enhancing the expression of extracellular enzymes, particularly β-CGTase by the recombinant *E. coli*.

## 1. Introduction

Cyclodextrin glycosyltransferase (CGTase, EC 2.4.1.19) is a carbohydrate-active enzyme (CAZy) that belongs to glycosyl hydrolase family 13 (http://www.cazy.org/). This enzyme catalyzes transglycosylation reactions, including hydrolysis, cyclization, coupling and disproportionation [1]. Through the cyclization reaction, CGTase catalyzes the conversion of starch into cyclodextrin, with well-characterized ones such as α, β and γ-cyclodextrins [2]. Cyclodextrin has a broad range of applications in the food [3], agriculture [4] and pharmaceutical [5] industries, as well as in environmental engineering [6], hence it is crucial to develop an efficient process for the synthesis of CGTase.

Due to the problems concerning low CGTase production at longer cultivation time by the natural host cells [7], the use of a genetically engineered strain is seen as the potential approach to fulfil the higher demand of cyclodextrin at an industrial scale. However, the issues of inclusion body formation, slow growth and inactivation of protein [8] has made the expression and production of CGTase from recombinant *Escherichia coli* even more challenging. In order to circumvent these obstacles, several strategies have been adopted that include codon usage optimization where codons are modified in a selected sequence to the high-frequency codons favored in the expression host [2]. Codon optimization is a synonymous mutation that modifies the nucleotide sequence of a gene without altering the amino acid sequence. 

Evidence showed that the choice of the synonymous codons can influence protein production by improving the correlation with the transfer RNA (tRNA) levels in the host [9] and changing the messenger RNA (mRNA) secondary structure [10]. The codons favored by the host cell are normally correlated with the large quantity of aminoacyl tRNAs [11] which guarantees the recycling of tRNA, hence resulting in an efficient gene translation. The presence of rare codons in the native gene could disrupt the translational efficiency due to the longer waiting time for a suitable aminoacyl tRNA [12]. In addition, rare codons could cause a slow elongation rate along the mRNA, resulting in ribosomal pause, hence causing the mRNA to be unstable [13]. In the worst case scenario, the rare codons could stimulate mRNA degradation [14]. 

It is also crucial to understand the genetic information of CGTase, so that a functional protein could be produced in a heterologous expression system. Several bioinformatics tools present in the ExPasy server, and in particular ProtParam [15], have been used to derive information about the stability, the total charge, the isoelectric point (pI) and hydrophobicity of β-CGTase.

The optimization of the cultivation process has been regularly employed to improve the expression of extracellular CGTase [16]. The use of inducers gives substantial advantages in altering the integrity of the cell wall of *E. coli* that will promote the secretion of extracellular recombinant enzyme into the culture medium. It is common that microbial cell membranes are very selective for the translocation of materials [17] in and out of the cell membrane. However, the rate and amount of materials transported across the cell membrane can be changed by altering the cell membrane permeability [18]. Glycine has been particularly shown to act as the best inducer in enhancing the secretion of recombinant β-CGTase as compared to Triton X-100 and xylose [19], even though all of these inducers were reported to play a role in increasing the permeability of cell membrane, which allowed protein translocation across the membrane. 

In a previous study, the individual supplementation of glycine into the culture media was shown to enhance the α-CGTase secretion from 2.4 to 32 U/mL at 48 h of cultivation [20]. It is even more impressive that the addition of glycine as an inducer did not cause apparent effects on bacterial cell viability [19], which indicates that the secretion of recombinant β-CGTase into the culture medium was not due to cell lysis. Since the optimization of either codon usage or glycine supplementation could enhance the secretion of enzyme into the culture medium, we are hypothesizing that the combined optimization could further improve the extracellular expression of β-CGTase even better than a single approach.

## 2. Results and Discussion

### 2.1. Construction of Recombinant E. coli for Expression of codon-Optimized cgt-BS Gene 

The codons for the native *cgt-BS* gene were optimized using the GenScript Rare Codon Analysis Tool software (https://www.genscript.com/tools/codon-frequency-table) to be more corresponding to the codon usage of *E. coli*, where the codon adaptation index (CAI) was increased from 0.67 to 0.78. Table 1 shows the codon usage of *E. coli* (as obtained from http://www.kazusa.or.jp/codon/; last accessed on 29 April 2019) as compared to the fraction of codons for both native (*cgt-BS*) and codon optimized (*cocgt-BS*) gene sequences. 

The replacements of several rare codons with those favored by *E. coli* were noted such as AGA(R) and CGA(R) to CGC(R) and AUA(I) to AUC(I). The major changes in frequencies of selected codons to those preferred by *E. coli* were also recorded such as CUG(L) from 2 to 27, UUA(L) from 31 to 13 and CCG(P) from 2 to 18. The changes in codons of β-cyclodextrin glycosyltransferase (CGT-BS) were balanced with the host preference without altering the amino acid sequences. In a previous study, the systematic codon optimization strategy was employed to further improve the expression of α-CGTase in *E. coli* with 2.79-fold increment following after IPTG induction at 48 h of fermentation [2]. In this study, the codon optimized *cocgt-BS* gene was constructed in pQE30xa and in frame with 6×-His tag to assist in protein purification using affinity chromatography. The expression vector was successfully constructed and verified by sequencing before it was transformed into *E. coli* JM109.

### 2.2. Prediction of Theoretical Physicochemical Properties of β-Cyclodextrin Glycosyltransferase (CGT-BS)

The physicochemical parameters of recombinant CGT-BS protein were analyzed using ExPASY’s ProtParam tool at http://web.expasy.org/protparam/ and compared with the selected *Bacillus* spp. CGTases proteins (Table 2). The positively charged amino acids for the selected CGTases proteins including CGT-BS were relatively similar, ranging between 46 and 52. The presence of low number of positively charged amino acids (Arg and Lys) as compared to negatively charged amino acids (Asp and Glu) in CGT-BS and other selected *Bacillus* spp. CGTases might contribute to the stability of these proteins. Arg was reported to act as suppressor of aggregation and destabilizer of proteins, while Lys have either little effect or might not contribute to the stability of proteins [21]. 

However, the formation of a salt bridge was suggested through the interaction of a positively charged amino acid with a negatively charged one, potentially contributing to protein conformational stability, especially when these two ionic groups are located adjacent to each other [22]. In addition, the aliphatic index of CGT-BS indicates a higher value and showed a close similitude to other selected CGTases proteins, hence indicating thermally stable CGTase proteins. Nonetheless, the instability index classified all selected CGTases including CGT-BS as stable proteins.

### 2.3. Optimization of Glycine Supplementation to Improve Extracellular Secretion of Recombinant β-CGTase

To further enhance the expression level of the extracellular recombinant β-CGTase from *E. coli*, the optimum glycine supplementation was determined using a central composite design (CCD). Glycine was found to cause morphological changes, such as an enlargement of spheroidal morphology in *E. coli*, as it is integrated into the nucleotide-activated peptidoglycan precursors, which leads to the increased permeability of host cell [23,24]. The glycine added into the culture medium could replace the alanine residues in the peptide component of the peptidoglycan of *E. coli* cell wall, resulting in a more loosely cross-linked peptidoglycan and therefore, enhancing the permeability of outer membrane of *E. coli* [25], without causing bacterial cell lysis [19,24]. 

A total of 20 experimental runs of different combinations of glycine supplementation were carried out in this study where the response was β-CGTase activity. The fifth column of Table 3 shows experimental values obtained in this study as compared to the values predicted by CCD (sixth column). β-CGTase activities measured were ranged from a minimum value of 3.109 U/mL to a maximum value of 65.733 U/mL. Based on Analysis of Variance (ANOVA) (Table 4), the model fit the data well and the model terms A, B and C corresponding to concentration of glycine, induction time and post-induction temperature, respectively were implied as significant factors that influence the expression of recombinant β-CGTase with p-values less than 0.05. In addition, *p*-value for ‘lack of fit’ showed greater than 0.05, hence indicated the ‘lack of fit’ of the model is insignificant. Therefore, the model was fit with the responses data collected and was desirable for further experiment. The multiple regression equation for the β-CGTase activity derived from the above three factors analyzed by Design Expert Software (Stat-Ease Inc., Minneapolis, MN, USA, Version 7) is as follows:

$$\beta \text{-}\mathrm{CGTase}=64.17+2.17A-3.15B+2.18C+3.90\mathrm{AB}-1.52\mathrm{AC}+0.4\mathrm{BC}-7.25A^{2}-9.96B^{2}-20.81C^{2}$$

where A, concentration of glycine; B, induction time; C, post-induction temperature.

The goodness of the model expressed by coefficient of determination (R^2^) was calculated as 0.9966. This explained 99.66% of the total variation for β-CGTase activity. Meanwhile, the Adjusted R^2^ was calculated to be 0.9936, indicating that only <1% of the total variation was not included in the model. The adjusted R^2^ also indicated that a good agreement was achieved between the response value and predicted value [26]. The predicted R^2^ of 0.9763 was in reasonable agreement with the adjusted R^2^. Therefore, the regression model was applied to calculate the predicted values (Table 3). From the results obtained, the optimum β-CGTase activity for the experimental and predicted values was achieved using 1.2 mM glycine, 37 °C post-induction temperature and 2 h induction time.

**Figure 1:**
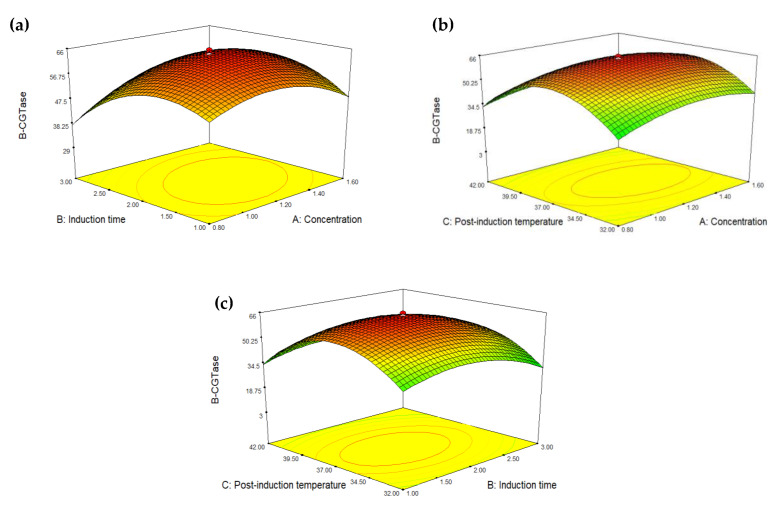
Effects of (**a**) glycine concentration vs. induction time, (**b**) glycine concentration vs. post-induction temperature, (**c**) post-induction temperature vs. induction time on the recombinant β-CGTase activities.

The relation between the concentrations of glycine and induction time on β-CGTase activity is shown in Figure 1a. The ANOVA showed that the effect of glycine concentration on induction time (AB) was the most significant factor on the expression of recombinant β-CGTase (*p* < 0.0001). The increased of glycine concentration until it reached 1.2 mM followed by the induction time at 2 h resulted in higher recombinant β-CGTase activity (65.733 U/mL). However, a reduction of the recombinant β-CGTase activity (29.961 U/mL) was observed at 3.68 h of induction time with fixed glycine concentration, hence indicating that induction time had greater effect than glycine concentration. When the induction time was kept constant at 2 h, the recombinant β-CGTase activity was 40.495 and 47.644 U/mL with the addition of 0.53 and 1.87 mM glycine, respectively (Table 3). At high inducer concentrations, the reduction in specific growth rate was critical that led to early stationary phase and cell death [27]. 

According to Li et al. [7], the extracellular secretion of the recombinant α-CGTase into the culture medium of *E. coli* was affected by glycine induction time, where the supplementation of glycine at the middle of the log phase of cell growth resulted in higher extracellular activity of the recombinant enzyme. A higher cellular energy level or carbon source was not only used for producing the recombinant protein, but also for the movement of proteins into the periplasm [28]. Therefore, the induction time between the early-log stage and middle-log phase where the carbon source is still available is suitable for the production of recombinant β-CGTase from *E. coli*. 

The effect of glycine concentration on post-induction temperature (AC) was also a significant factor (*p* = 0.0246) that influenced the expression of the extracellular recombinant β-CGTase (Figure 1b). A higher glycine concentration at higher post-induction temperature resulted in an increment of the recombinant β-CGTase production until it reached the optimum level which was at 37 °C post-induction temperature and 1.2 mM of glycine concentration. Any further increase beyond this point led to reduction of the recombinant β-CGTase activity. When the induction time was kept constant at 2 h with 1.2 mM glycine concentration, the recombinant β-CGTase activity was 8.469 and 3.109 U/mL at 28 °C and 45 °C post induction temperature, respectively. 

It has been stated that the number of ribosomes per cell and level of ribosomal ribonucleic acid (rRNA) remained constant in the temperature range of 23–42 °C [29]. The polypeptide chain elongation rate would also increase with an increase in temperature, resulting in higher synthesis of proteins. However, by further increasing the temperature, the microorganism spends a lot of energy for maintenance. On the other hand, the transport of nutrients is also hindered at lower temperature [30]. Nevertheless, the interaction between induction time and post-induction temperature gave insignificant effect to the expression of recombinant β-CGTase (*p* = 0.5044), which was greater than 0.05 (Figure 1c). Lo et al. [26] stated that the interactions among the cultivation conditions would not give a significant influence towards the expression of the enzyme.

**Figure 2:**
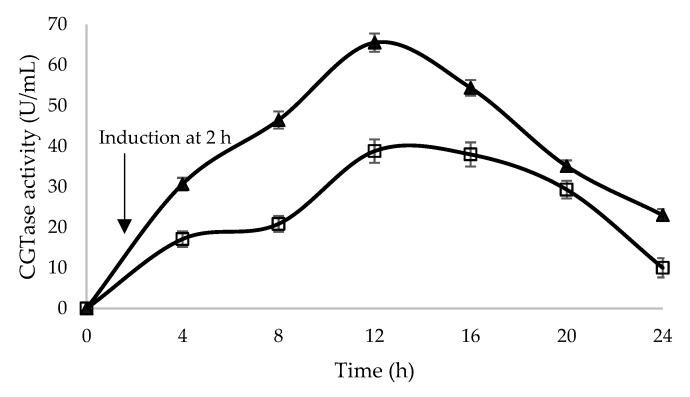
*E. coli* harboring pQEcoCGT-BS were grown in Terrific Broth (TB) media without glycine (□) and with glycine supplementation at the optimized conditions (▲) which were at post-induction temperature of 37 °C, glycine concentration of 1.2 mM and induction at 2 h of cultivation as indicates by arrow.

To confirm the model’s adequacy for predicting the maximum β-CGTase activity, the experiment was carried out by using optimum conditions (1.2 mM glycine, 37 °C post-induction temperature and 2 h induction time) with a desirability of 0.965. Based on the results obtained, in comparison with the predicted value, there was relatively small error which was only 2%. Therefore, a good agreement was achieved between the predictive and actual values of β-CGTase activity at the optimum conditions, giving high validity of the model. The supplementation of glycine into the culture medium enhanced the extracellular secretion of β-CGTase activity by *E. coli* carrying pQEcocgt-BS as compared to the culture without glycine (Figure 2). 

With the addition of glycine using the optimized feeding strategy as discussed earlier, the β-CGTase activity increased gradually and reached its highest secretion of 65.524 U/mL at 12 h of cultivation, which was increased by 1.7-fold in comparison to a single approach of codon usage optimization and 5.6-fold as compared to the wild type strain when using soluble starch as a substrate [31]. In a different study by Ding et al. [17], the secretion of recombinant α-CGTase into the culture medium of *E. coli* was improved by adding 0.3% glycine, resulted in 12.89 U/mL of activity, which was 15 times higher as compared to the culture without glycine.

### 2.4. Purification of Recombinant β-CGTase and Its Kinetic Parameters

The recombinant β-CGTase was purified from the 12 h culture induced with 1.2 mM glycine at 2 h induction time at 37 °C. The recombinant β-CGTase carried an N-terminal fusion peptide containing a 6×-His sequence that has metal-binding affinity. The enzyme was purified through a combination of diafiltration and nickel-nitrilotriacetic acid (Ni-NTA) affinity chromatography, which resulted in increased purity to 20.1-fold. The protein was then concentrated using ultrafiltration where the purity was increased to 23.8-fold (Table 5). However, by taking into account the theoretical extinction coefficient for the oxidized and reduced CGT-BS protein of 142,810 and 142,560 M^−1^·cm^−1^, respectively (Table 2) with the absorbance of the purified (ultrafiltration) protein fraction was 0.109, the purified protein concentration was calculated to be 0.06 mg/mL. Nonetheless, the purity of codon optimized protein (*cocgt-BS*) obtained in this study was not much different with the resultant recombinant *cgt-BS* protein (non-optimized codon) in the previous study [19]. Similarly, the results of sodium dodecyl sulfate–polyacrylamide gel electrophoresis (SDS-PAGE) also showed the expected band size of the purified protein at approximately 80 kDa (Supplementary Figure S1), in correlation with the theoretical molecular weight predicted using ExPASY’s ProtParam online tool (Table 2). 

**Figure 3:**
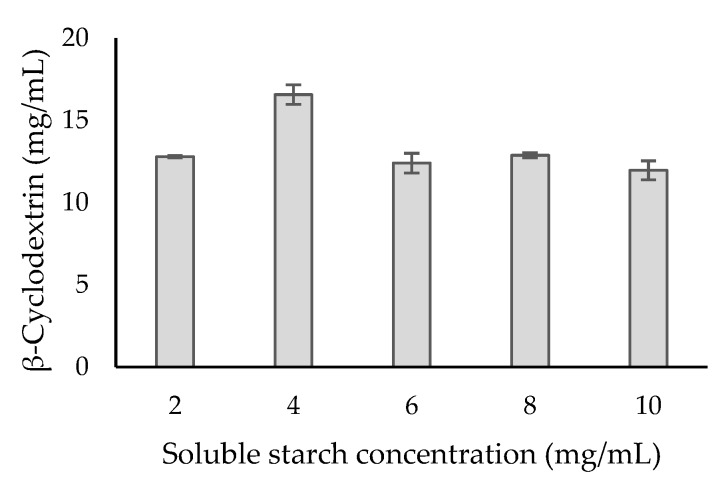
Production of β-cyclodextrin by purified β-CGTase using different concentrations of soluble starch. Standard deviations are shown as bars and the deviation from the mean is below 5%.

**Figure 4:**
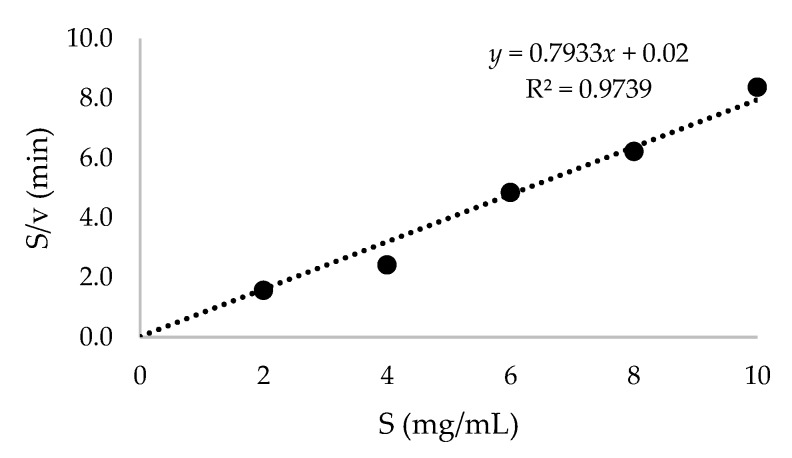
Hanes–Woolf plot to identify *K*_m_ and *V*_max_ which were used in the Michaelis–Menten equation. S: substrate, v: velocity.

A different range of soluble starch concentrations was then tested to produce β-cyclodextrin by recombinant β-CGTase. A maximum β-cyclodextrin of 16.57 mg/mL was achieved by using 4 mg/mL of soluble starch (Figure 3). However, the production of β-cyclodextrin was reduced and attained almost a similar concentration with an increased soluble starch concentration. At this point, the enzyme could probably reach saturation with the substrate [32]. The cyclization reaction of β-CGTase was then described by Michaelis–Menten kinetic parameters (Figure 4) to evaluate the performance of recombinant β-CGTase for β-cyclodextrin production [33,34]. The *V*_max_ and *K*_m_ values were evaluated to be 1.25 mg/mL/min and 0.025 mg/mL, respectively. A small value of *K*_m_ could indicate a high affinity for the substrate [35] and it could achieve its highest catalytic efficiency at minimum concentration of soluble starch. 

### 2.5. Characterization of Recombinant β-CGTase

**Figure 5:**
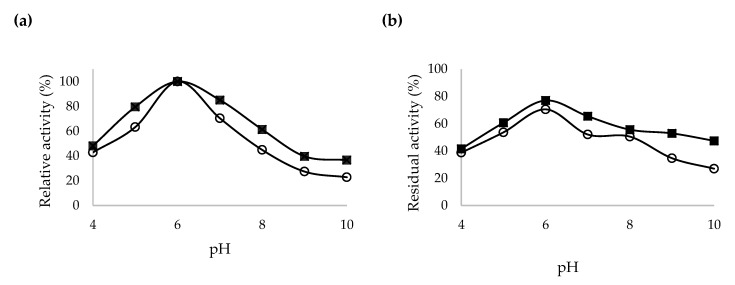
Properties of the recombinant β-CGTase produced by *E. coli* harboring pQEcoCGT-BS; (**a**) Optimal pH and (**b**) pH stability of the crude (○) and purified (■) β-CGTase.

The effects of pH and pH stability on the crude and purified codon optimized β-CGTase activities were studied and presented in Figure 5a,b, respectively. The purified β-CGTase was highly tolerant in the pH range of 5–7, with relative activity equivalent to 80% and more. Nonetheless, both the crude and purified β-CGTases showed an optimum activity at pH 6. Extreme conditions at pH 4 and pH 8–10 gave critical effects on both crude and purified β-CGTases relative activity with reduction of more than 52%. After incubating the crude and purified β-CGTases with different pH buffer for 30 min, the purified β-CGTase showed more than 50% residual activity at pH 5–9. Meanwhile, crude β-CGTase showed large reduction of its residual activity at the same pH range by 30–65%. An almost similar result was obtained by Ong et al. [35] where the purified recombinant CGTase G1 was found to be stable over a wide range of pH from 5 to 9 with a gradual loss of activity at acidic pH.

**Figure 6:**
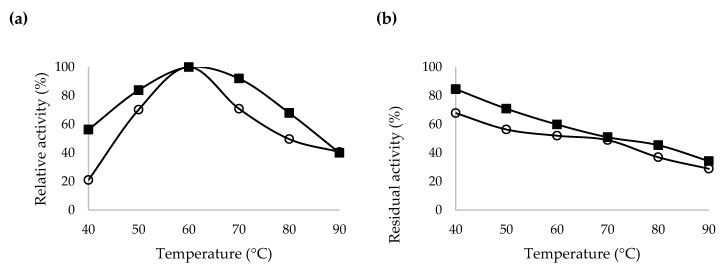
Properties of the recombinant β-CGTase produced by *E. coli* harboring pQEcoCGT-BS; (**a**) Temperature profile and (**b**) temperature stability of the crude (○) and purified (■) β-CGTase.

The effects of temperature and temperature stability on crude and purified β-CGTase activities are presented in Figure 6a,b, respectively. The purified β-CGTase could be classified as thermo-tolerant, able to maintain a relative activity of more than 84% at 50–70 °C. However, the crude β-CGTase showed a lower temperature tolerance where the relative activity was reduced to approximately 70% at the same temperature ranges. However, the optimum temperature for both crude and purified β-CGTases was the same at 60 °C. 

Following that, the crude and purified β-CGTases were incubated for 30 min with soluble starch at different temperatures ranged from 40 to 90 °C. The residual activity of the purified β-CGTase was reduced to 60% at 60 °C, but the residual activity of the crude β-CGTase was slightly lower at the same temperature which was at 52%. In a previous study, the purified α-CGTase retained 50% of its initial cyclization activity after incubation for approximately 8 h at 40 °C, 1.25 h at 45 °C and 0.5 h at 50 °C [7]. The high stability of the purified β-CGTase produced from this study with respect to pH and temperature could makes this recombinant enzyme potentially useful in various applications. However, more characterization studies need to be carried out in the future prior to its application at an industrial scale.

## 3. Materials and Methods 

### 3.1. Bacterial Strain and Plasmid

*cgt-BS* gene from a previous study [36] was mutated with codon usage optimization. *Escherichia coli* JM109 [endA1, recA1, gyrA96, thi, hsdR17 (rk-,mk+), relA1, supE44, D (lac-proAB), F’ (tra D36, pro AB, lacIqZ Δ M15)] from Promega (Madison, WI, USA) was used as a host strain and plasmid pQE30xa (QIAGEN, Hilden, Germany) was used as an expression vector.

### 3.2. Codon Optimization of cgt-BS Gene

The codons of the *cgt-BS* gene with the size of 2460 bp originally from *Bacillus* sp. NR5 UPM (GenBank accession number: HQ876173.1) were optimized based on the codon preference of *E. coli* by referring to a codon table containing fractional preference for each codon equal to that found in the genome of *E. coli*. By using the GenScript Rare Codon Analysis Tool software (https://www.genscript.com/tools/codon-frequency-table), the codons were optimized without compromising the encoded amino acid sequences functional for protein expression. The synthesis of the codon-optimized gene was performed by Apical Scientific Sdn. Bhd., Malaysia.

### 3.3. Construction of the Expression System

The amplified PCR fragment encoding the codon optimized *cgt-BS* gene was ligated into pQE30xa vector, designated as pQEcoCGT-BS. The amplification of *cocgt-BS* gene was performed under standard PCR conditions with 1 μg of genomic DNA, 200 ρmol of each forward and reverse primer, 0.01 μL of *Taq* DNA polymerase in 1× reaction buffer and 0.2 mM of dNTPs in a final volume of 20 μL. The specific primers designed for amplification of *cocgt-BS* gene used for construction of pQEcoCGT-BS expression system were coCGTF 5′-GGATCCCTTCTGTAAATTGACCAACCGG-3′ and coCGTR 5′-AAGCTTTTACCAGTTGATCATTACGGTA-3′ for forward and reverse primer, respectively. The forward and reverse primers were designed to contain the restriction sites *Bam*HI and *Hin*dIII (underlined), respectively. The amplified DNA fragment was purified after performing agarose gel electrophoresis using Hi Yield Gel/PCR DNA Mini Kit (Real Biotech Corporation, Taipei, Taiwan) and digested with *Bam*HI and *Hin*dIII before ligation into pQE30xa. The ligation products were subsequently introduced into *E. coli* JM109. Luria-Bertani (LB)-ampicillin (100 μg/mL) was used to plate out the transformation mixtures. After overnight growth at 37 °C, colonies were chosen for insert confirmation. 

### 3.4. Prediction of Physicochemical Characteristics

Multiple physicochemical characteristics of the CGT-BS protein were evaluated by ExPASY’s ProtParam online tool at http://web.expasy.org/protparam/ [15] including theoretical isoelectric point (pI), number of positively and negatively charged amino acids, extinction coefficient (EC), instability index (II), aliphatic index (AI), grand average of hydropathicity (GRAVY) and total number of atoms (TNA), in comparison to other CGTases from selected *Bacillus* spp.

### 3.5. Medium and Culture Conditions 

Recombinant *E. coli* JM109 harboring pQEcoCGT-BS was inoculated into LB broth containing 100 μg/mL ampicillin for preparation of inoculum. The inoculum flask was incubated overnight at 37 °C in an incubator shaker, agitated at 200 rpm until an optical density of 1.5 at 600 nm was obtained. The inoculum size of 10% *v*/*v* was adjusted and standardized throughout the experiments. The production medium was prepared using Terrific Broth (TB) supplemented with 100 μg/mL ampicillin for the preparation of crude recombinant β-CGTase, according to the design of experiment carried out using central composite design (CCD) as stated in Section 3.6. The culture was incubated at 37 °C, agitated at 200 rpm for 24 h and sampled every 4 h. For estimation of cell growth, the pellets were appropriately diluted, and the optical density was measured at 600 nm by using spectrophotometer (Hitachi U-2900, Hitachi, Japan).

### 3.6. Design of Experiments for Optimum Condition of Glycine Supplementation

Experimental design was carried out using Design Expert Software (Stat-Ease Inc., Minneapolis, MN, USA, Version 7). The results obtained from the CCD was subjected to the statistical analysis to identify the variables that had significant effect on the response which was β-CGTase activity. Three parameters which were the concentration of glycine (mM), time of induction (h) and post-induction temperature (°C) consisting 20 runs (14 combinations with 6 replications of the center points and axial points of α equals to 1.68) were carried out in this study. The value set for each parameter is given in Table 3. The range of each parameters were based on previous study where the glycine supplementation was optimized using one factor at a time approach [19]. All values were tested in order to get the maximum production of recombinant β-CGTase. Expression of β-CGTase was then confirmed by measuring its activity following 12 h of fermentation with culture condition stated in Section 3.5 and with the optimal glycine supplementation. For the determination of enzyme expression profile, cell-free supernatant was collected by centrifuging the fermentation broth at 14,500× *g* for 10 min at 4 °C. All runs were conducted in triplicate and the significance value of the experimental data was analyzed using ANOVA test to determine factors having a significant effect (*p* ≤ 0.05). The validation of the response surface model was done in triplicate under the predicted optimum conditions.

### 3.7. Purification of Recombinant β-CGTase

The recombinant β-CGTase was subjected to AKTA Flux tangential flow filtration system (GE Healthcare, New York, NY, USA) using a 30 kDa molecular weight cut-off (MWCO) membrane, equilibrated with 10 mM imidazole. Affinity purification was then performed on a Ni-NTA column on an AKTA Avant chromatography system (GE Healthcare) according to the manufacturer’s instructions. The sample was incubated for 30 min in the column, followed by washing with buffer containing 20 mM imidazole (pH 8.0). Elution buffer containing 250 mM imidazole (pH 8.0) was used to elute the bound enzyme. The fractions with maximum activity were pooled together and Amicon ultrafiltration membrane kit (30 kDa MWCO membrane) was used to concentrate the samples. The samples were assayed for β-CGTase activity and protein concentration.

### 3.8. Assay of β-CGTase Activity 

The β-CGTase activity was measured using phenolphthalein assay [37]. Reaction mixture containing 1 mL of 40 mg of soluble starch in 100 mM phosphate buffer (pH 6.0) and 0.1 mL of enzyme solution was incubated at 60 °C for 10 min in a water bath. Then, 3.5 mL of 30 mM NaOH was added to stop the reaction. Subsequently, 0.5 mL of 0.02% (*w*/*v*) phenolphthalein in 5 mM Na_2_CO_3_ solution was added to the reaction mixture and mixed well. After 15 min, the reduction in color intensity was measured at 550 nm. Blanks lacking the CGTase were analyzed simultaneously with each batch of samples. As a standard, the soluble starch and enzyme were replaced with 0.5 mg of β-cyclodextrin and 0.1 mL of water, respectively. A calibration curve was made using β-cyclodextrin in 100 mM phosphate buffer, pH 6.0. One unit of enzyme activity was defined as the amount of enzyme that formed 1 µmol β-cyclodextrin per min under the conditions defined above.

### 3.9. Molecular Mass Determination of β-CGTase

Protein concentration was measured according to Lowry et al. [38] with bovine serum albumin as a standard. The protein size was analyzed using sodium dodecyl sulfate–polyacrylamide gel electrophoresis (SDS-PAGE) at constant voltage of 100 V for 1.5 h at room temperature until the band was migrated sufficiently. Gel (8.3 cm × 7.3 cm) was run according to the method of Laemmli [39]. The concentrations of the stacking and resolving gels were 5% and 12%, respectively. Appropriately diluted protein samples (30 μg) with 4× marker containing 50 mM Tris–HCl (pH 6.8), 40% (*w*/*v*) of glycerol, 10% (*w*/*v*) of SDS, 5% (*v*/*v*) of mercaptoethanol and 0.05% (*w*/*v*) of bromophenol blue were boiled for 5 min before applied into each lane. The lane was formed by a 10-well comb (Mini-PROTEAN^®^, Bio-Rad, Hercules, CA, USA) and the molecular weight marker (PageRuler™ Unstained Protein Ladder) from Thermo Scientific, Waltham, MA, USA was used as a standard. The gel was stained with Coomassie Brilliant Blue R-250 (Fisher Scientific, Waltham, MA, USA).

### 3.10. Characterization of Crude and Purified Codon Optimized Recombinant β-CGTase

The measurement of the optimum pH for the crude and purified recombinant β-CGTase was done by reacting 0.1 mL enzyme with 1 mL of 40 mg of soluble starch dissolved in different buffers at pH 4–9 [40]. The phosphate buffer (0.1 M), pH 6 used in the β-CGTase assay was replaced with the following buffers: sodium acetate buffer, 0.1 M (pH 4–5), potassium phosphate buffer, 0.1 M (pH 6–8) and glycine-NaOH buffer, 0.1 M (pH 9). Meanwhile, the effect of pH stability on crude and purified recombinant β-CGTase was determined by incubating 0.1 mL of enzyme with 0.2 mL of different buffers at pH 4–9, without substrate at 50 °C for 30 min.

On the other hand, the optimum temperature for the crude and purified recombinant β-CGTase was determined by incubating the reaction mixture of β-CGTase assay in 0.1 M phosphate buffer, pH 6.0 at different temperatures ranging from 40 to 80 °C for 10 min [40]. Meanwhile, the effect of temperature on the stability of crude and purified recombinant β-CGTase was determined by incubating 0.1 mL of enzyme with 0.2 mL of 0.1 M phosphate buffer (pH 6.0) without substrate for 30 min at different temperatures, ranging from 40–90 °C. For both effects of pH and temperature, the reaction was carried out according to the method of β-CGTase assay as described above [37].

### 3.11. Cyclodextrin Production and Kinetic Parameters of Purified β-CGTase

The kinetic parameters (*K*_m_ and *V*_max_) for the pure enzyme were determined by incubating 0.5 U/mL of purified β-CGTase in 0.1 M phosphate buffer (pH 6.0) at various concentrations of soluble starch solution, ranging from 2 to 10 mg/mL at 60 °C for 30 min. The values of *K*_m_ and *V*_max_ were analyzed by linear regression with the linear transformation (Hanes-Woolf plot) of the Michaelis–Menten equation using an Excel curve-fitting program (Microsoft Excel 2010, San Francisco, CA, USA) [41]. The concentrations of β-cyclodextrin in the final sample were determined by Refractive Index–High Performance Liquid Chromatography (RI-HPLC) (Shimadzu, Kyoto, Japan), using a Lichrospher-NH_2_ column (Merck, Darmstadt, Germany) eluted with 65:35 acetonitrile–water at 1 mL/min.

## 4. Conclusions

The combined strategy of codon usage modification and optimization of glycine supplementation has been shown to greatly improve the expression of extracellular recombinant β-CGTase as compared to a single approach. The adaptation of rare codons of the *cgt-BS* gene to the preferred ones in *E. coli* is important to ensure an efficient expression of functional protein, as demonstrated by a CAI of 0.78. With the help of an in-silico tool, the recombinant CGT-BS protein was determined as a stable protein. In addition, the optimized glycine feeding into the cultivation medium at the concentration of 1.2 mM, induced at 2 h induction time at 37 °C post-induction temperature altogether managed to enhance the recombinant β-CGTase activity up to 2.2-fold at 12 h of cultivation as compared to un-optimized conditions. This combined approach could be potentially used to further enhance the expression of recombinant protein at the upstream and downstream levels.

## Figures and Tables

**Table 1:** Codon preference in *E. coli*, codon usage in the native gene (*cgt-BS*) and the optimized synthetic gene (*cocgt-BS*).

Amino Acid	Codon	Relative Frequency	Frequency		Amino Acid	Codon	Relative Frequency	Frequency	
		*E. coli*	*cgt-BS*	*cocgt-BS*			*E. coli*	*cgt-BS*	*cocgt-BS*
Ala (A)	GCA	0.22	11	7	Leu (L)	CUA	0.03	5	0
	GCC	0.25	8	9		CUC	0.10	4	6
	GCG	0.34	5	16		CUG	0.55	2	27
	GCU	0.19	9	1		CUU	0.10	9	5
Arg (R)	AGA	0.04	3	0		UUA	0.11	31	13
	AGG	0.03	1	0		UUG	0.11	9	9
	CGA	0.05	5	0	Lys (K)	AAA	0.76	22	27
	CGC	0.37	4	12		AAG	0.24	10	5
	CGG	0.08	1	2	Met (M)	AUG	1.00	15	15
	CGU	0.42	8	8	Phe (F)	UUC	0.49	11	14
Asn (N)	AAC	0.61	34	41		UUU	0.51	24	21
	AAU	0.39	36	29	Pro (P)	CCA	0.20	7	4
Asp (D)	GAC	0.41	11	20		CCC	0.10	3	2
	GAU	0.59	37	28		CCG	0.55	2	18
Cys (C)	UGC	0.57	1	4		CCU	0.16	7	5
	UGU	0.43	3	0	Ser (S)	AGC	0.27	16	19
Gln (Q)	CAA	0.31	22	10		AGU	0.13	11	8
	CAG	0.69	10	22		UCA	0.12	16	14
Glu (E)	GAA	0.70	22	21		UCC	0.17	6	9
	GAG	0.30	9	10		UCG	0.13	4	14
Gly (G)	GGA	0.09	13	3		UCU	0.19	14	3
	GGC	0.40	17	22	Thr (T)	ACA	0.30	17	11
	GGG	0.13	13	7		ACC	0.43	14	26
	GGU	0.38	16	27		ACG	0.23	14	13
His (H)	CAC	0.48	7	7		ACU	0.21	11	6
	CAU	0.52	10	10	Trp (V)	UGG	1.00	15	15
Ile (I)	AUA	0.07	12	0	Tyr (Y)	UAC	0.47	20	19
	AUC	0.46	13	30		UAU	0.53	28	29
	AUU	0.47	24	19	Val (V)	GUA	0.17	18	10
						GUC	0.20	11	18
						GUG	0.34	5	11
						GUU	0.29	19	14

**Table 2:** Physicochemical parameters of CGT-BS (this study) in comparison to the other CGTases from selected *Bacillus* spp., computed using ExPASY’s ProtParam tool.

Description	Accession Number	Theoretical MW (Da)	pI	R+	R−	EC ^a^ (M^−1^·cm^−1^)	EC ^b^ (M^−1^·cm^−1^)	II	Stability	AI	GRAVY	TNA
*Bacillus* sp. NR5 UPM cyclodextrin glycosyltransferase (*cgt*) gene *	HQ876173.1	80,622.34	4.81	48	76	142,810	142,560	24.57	stable	75.36	−0.405	11,108
*B. circulans* strain 251 *cgt* gene	X78145.1	77,309.28	6.08	52	58	120,795	120,670	22.72	stable	74.05	−0.250	10,710
*B. licheniformis cgtA* gene for cyclomaltodextrin glucanotransferase (CGTase)	X15752.1	78,002.68	5.57	48	59	124,805	124,680	18.17	stable	71.75	−0.243	10,785
*Bacillus* sp. (strain no. 38-2) cyclomaltodextrin glucanotransferase (CGTase) gene	M19880.1	78,249.29	5.41	49	63	133,285	133,160	25.23	stable	75.76	−0.286	10,826
*Bacillus* sp. strain Y112 cyclodextrin glycosyltransferase precursor (cgtase) gene	KX579963.2	80,168.90	4.23	46	111	129,735	129,610	30.92	stable	73.39	−0.483	10,983
*Bacillus* sp. BL-31 cyclodextrin glucanotransferase (*cgt*) gene	EF363797.1	80,096.84	4.24	46	110	129,735	129,610	30.65	stable	73.39	−0.478	10,974

* This study. Theoretical isoelectric point (pI), number of positively charged amino acids − Arg + Lys (R+); number of negatively charged amino acids − Asp + Glu (R−); extinction coefficient (EC) (^a^: assuming all pairs of Cys residues form cystines, ^b^: assuming all Cys residues are reduced); instability index (II); aliphatic index (AI); grand average of hydropathicity (GRAVY); total number of atoms (TNA).

**Table 3:** Central composite design (CCD) matrix, the predicted and experimental values obtained for the expression of codon optimized CGTase (*cocgtBS*) from recombinant *E. coli.*

Run	Concentration (mM)	Induction Time (h)	Post-Induction Temperature (°C)	β-CGTase Activity (U/mL)	
				Experimental	Predicted
1	1.60	1.00	42.0	23.47	23.47
2	1.60	3.00	32.0	34.23	32.38
3	1.20	2.00	37.0	64.76	64.17
4	1.20	2.00	37.0	63.88	64.17
5	1.60	3.00	42.0	24.37	25.77
6	1.20	2.00	37.0	62.72	64.17
7	1.20	2.00	37.0	65.73	64.17
8	1.20	3.68	37.0	29.96	30.70
9	1.20	2.00	37.0	63.97	64.17
10	1.60	1.00	32.0	30.18	31.67
11	1.20	2.00	37.0	63.83	64.17
12	0.80	3.00	32.0	16.61	17.19
13	1.20	0.32	37.0	42.88	41.31
14	0.80	3.00	42.0	17.57	16.66
15	1.20	2.00	28.6	8.47	9.11
16	0.80	1.00	32.0	32.92	32.10
17	1.20	2.00	45.4	3.11	1.65
18	1.87	2.00	37.0	47.64	47.31
19	0.80	1.00	42.0	27.55	29.98
20	0.53	2.00	37.0	40.50	40.01

**Table 4:** Analysis of Variance (ANOVA) for response surface quadratic model for the expression of extracellular recombinant CGTase (*cocgtBS*) from recombinant *E. coli.*

Source	Sum of Squares	Degree of Freedom (df)	Mean Square	F Value	*p*-Value (Probability > F)	Significant Term Based on *p*-Value
Model	7839.26	9	871.03	329.64	<0.0001	*Significant*
A	64.26	1	64.26	24.32	0.0006	
B	135.82	1	135.82	51.40	<0.0001	
C	64.98	1	64.98	24.59	0.0006	
AB	121.96	1	121.96	46.16	<0.0001	
AC	18.47	1	18.47	6.99	0.0246	
BC	1.27	1	1.27	0.48	0.5044	
A^2^	758.03	1	758.03	286.88	<0.0001	
B^2^	1428.85	1	1428.85	540.75	<0.0001	
C^2^	6229.12	1	6229.12	2357.42	<0.0001	
Residual	26.42	10	2.64			
Lack of fit	21.29	5	4.26	4.14	0.0724	*Not significant*
Pure error	5.14	5	1.03			
Cor total	7865.69	19				
R^2^	0.9966					
Adjusted R^2^	0.9936					

**Table 5:** Purification yield of recombinant codon optimized β-CGTase (*cocgtBS*) from *E. coli* following induction with 1.2 mM glycine at 2 h of fermentation at 37 °C.

Purification Step	Volume (mL)	Total Activity (U/mL)	Total Protein (mg/mL)	Specific Activity (U/mg)	Purification Fold	Purification Yield (%)
Crude	1000	77.1	16.2	4.8	1.0	100.0
Diafiltration	250	65.6	10.6	6.2	1.3	85.2
Affinity Chromatography	9	19.1	0.2	95.6	20.0	24.8
Ultrafiltration	3	11.0	0.1	113.4	23.8	14.3

## Supplementary Materials

Supplementary materials can be found at https://www.mdpi.com/1422-0067/21/11/3919/s1.
