# Supplementary material for: Combined Optimization of Codon Usage and Glycine Supplementation Enhances the Extracellular Production of a β-Cyclodextrin Glycosyltransferase from Bacillus sp. NR5 UPM in Escherichia coli
Source: Int J Mol Sci. 2020 May 30;21(11):3919. doi: 10.3390/ijms21113919 (PMC7313058; doi:10.3390/ijms21113919)
Supplement: Supplementary file 1 [file ijms-21-03919-s001.pdf]

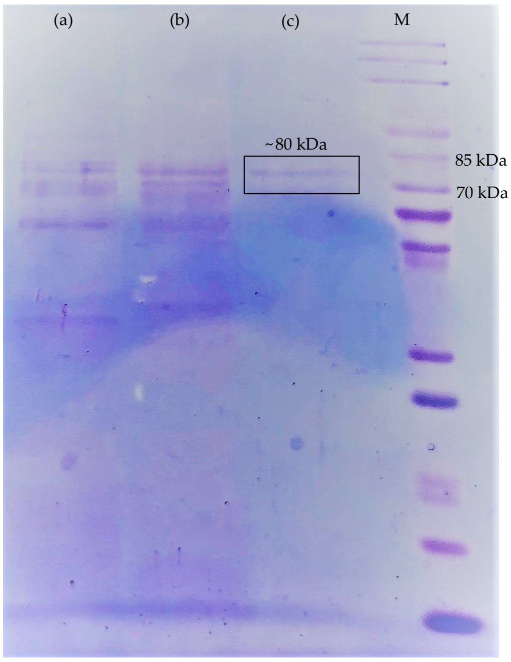

Figure S1. SDS-PAGE analysis of the crude and purified recombinant proteins: (**a**) crude recombinant protein; (**b**) recombinant protein following diafiltration; (**c**) purified protein following affinity chromatography and concentrated via ultrafiltration. All protein samples were loaded with the total concentration of 30 μg for each well. M: PageRuler unstained protein leader (Thermo Fisher Scientific, USA).
